# The role of nucleating agents in high-pressure-induced gamma crystallization in isotactic polypropylene

**DOI:** 10.1007/s00396-014-3445-z

**Published:** 2014-11-13

**Authors:** Przemyslaw Sowinski, Ewa Piorkowska, Severine A. E. Boyer, Jean-Marc Haudin, Kinga Zapala

**Affiliations:** 1 Centre of Molecular and Macromolecular Studies, Polish Academy of Sciences, Sienkiewicza 112, 90 363 Lodz, Poland; 2 Department of Physics and Mechanics of Materials, P PRIME Institute–ISAE-ENSMA, UPR CNRS 3346, 1 Avenue Clément Ader, 86961 Futuroscope Chasseneuil, France; 3 Centre for Material Forming, MINES ParisTech, PSL–Research University, UMR CNRS 7635, 1 Rue Claude Daunesse, 06904 Sophia Antipolis, France

**Keywords:** Crystallization, High pressure, Nucleating agents, Gamma nucleation, Isotactic polypropylene

## Abstract

Nucleation of the γ-form in isotactic polypropylene (PP) under high pressure was investigated. Three nucleating agents were used to nucleate crystallization of PP under atmospheric pressure: commercial Hyperform HPN-20E from Milliken Chemical, poly(tetrafluoroethylene) particles nucleating the α-form, and calcium pimelate nucleating the β-form. Crystallization of neat PP and PP with addition of 0.2 wt% of the nucleating agents was studied. Specimens were either kept at 200 °C under pressure of 200 MPa for time ranging from 2 min to 4 h or for 15 min under pressure ranging from 1.3 to 300 MPa. After cooling to ambient temperature and releasing the pressure, the specimens were analyzed by DSC, WAXD, and PLM to have an insight into the structure and to determine a crystallinity level and contents of crystallographic forms. Both α-nucleating agents strongly nucleated crystallization of PP under high pressure in the γ-form, whereas the β-nucleating agent had only a slight effect. The results show the possibility to use nucleating agents to nucleate the γ-form of PP under high pressure.

## Introduction

Isotactic polypropylene (PP) can crystallize in three crystalline forms: monoclinic alpha (α), trigonal beta (β), and orthorhombic gamma (γ) or in the mesomorphic form. The mesophase, called “smectic”, forms at very large undercooling (Δ*T*), reached via fast quenching. The monoclinic α-form crystallizes under common processing conditions. The important feature of the α-modification is so-called cross-hatched morphology which results from lamellar branching of crystallographic origin involving self-epitaxy on (010) crystallographic plane; “daughter” lamellae are tilted at an angle of 80 or 100 ° to “mother” lamellae.

The β-phase can be nucleated by special nucleating agents [1]. It was also reported that shear [eg., 2], and temperature gradient during zone solidification [3] enhance the formation of the β-phase. However, no such effect was found during crystallization of PP in a constant steady-state temperature gradient [4].

The orthorhombic γ-modification is unique because of a nonparallel chain arrangement. The γ-form crystals are formed by bilayers composed of parallel helices [5, 6] with the direction of the chain-axis in adjacent bilayers tilted at an angle of 80 or 100 ° to each other [5–7], that is the same as between mother and daughter lamellae of the α-modification. Mechanical properties and plastic deformation mechanism of both β and γ-forms differ from those of the α-modification [8, 9]. The plane-strain and uniaxial compression tests demonstrated that γ-PP exhibited higher modulus, higher yield stress and flow stress, and slightly lower ultimate strain than α-PP [9]. The γ-phase seldom forms during crystallization of PP homopolymer under atmospheric pressure (*P*_atm_) [10, 11]. The formation of the γ-phase was observed in low molecular weight PP [12–15], and in the presence of chain defects or chemical heterogeneities resulted from either atacticity [16, 17] or copolymerization with 1-olefine co-units [12, 16, 18–23]. It was also found that the formation of the γ-phase was enhanced by small Δ*T* and by nucleating agents [24]. It is long known that crystallization of the γ-form of highly stereoregular PP is facilitated by increase of pressure (*P*) [25, 26], although it requires also appropriate high temperature (*T*). It is well known that the increase of pressure increases temperatures of phase transitions in polymers. Based on their extensive experimental data, Mezghani and Phillips [26] determined equilibrium melting temperature, (*T*_*m*_^*0*^) of the γ-modification and constructed a temperature-pressure phase diagram for the α and γ-forms.

The formation of the high-pressure γ-phase at small Δ*T* suggests the importance of heterogeneous nucleation. To clarify that point, Zapala et al. [27] studied crystallization in PP droplets under high *P*, in that region of the phase diagram where the γ-phase is stable, that is at 200 °C and 200 MPa. The γ-phase was found to form predominantly only in the droplets sufficiently large to contain the most active heterogeneities, which were able to nucleate PP crystallization in the usual α-form under *P*_atm_, and crystallized in the same *T* range as bulk PP. In the smaller droplets, which solidified under *P*_atm_ at markedly lower *T*, the γ-phase did not form under high *P*. This result indicates possibility to nucleate crystallization of the γ-phase by means of nucleating agents.

The search for nucleating agents of PP is an old story. Since the pioneering works of Beck [28] and Binsbergen [29], a huge number of systems have been tested. To give a scientific basis to this search, Binsbergen [30] proposed a theory of heterogeneous nucleation in polymers, which illustrates the role of surface energies. Unfortunately, this theory is not easy to use and cannot explain all the effects observed. A fundamental explanation of heterogeneous nucleation was proposed by the group of Lotz, which developed an integrated theory of epitaxial interaction between nucleating agent and polymer [31, 32]. Nevertheless, the concept of epitaxy is not sufficient for finding technically suitable nucleating agents for PP as well as for other polymers. The overall efficiency of nucleation results from both epitaxy quality and dispersion quality. Even with a deep understanding of crystallographic details, the practical efficiency has to be checked experimentally. A more absolute criterion is obtained by comparison with self-nucleation [33].

In a recent article, Gahleitner et al. [34] reviewed the known systems for iPP nucleation. The nucleating agents for α-PP can be inorganic (talc, wollastonite, and mica) or organic. The by far larger class of organic nucleants can be subdivided into three categories: particulate nucleating agents like carboxylic acid salts (benzoates and aromatic organophosphates), soluble nucleating agents like sorbitols and trisamides, and polymeric nucleating agents like poly(tetrafluoroethylene) (PTFE). Among the most used nucleation agents for β-PP are γ-quinacridone, *N*,*N*′-dicyclohexylnaphthalene-2,6-dicarboxamide, calcium pimelate, or suberate.

In order to check if the use of nucleating agents could actually enhance the crystallization in the γ-crystal form under high *P*, two α-nucleating agents were selected among the existing ones and a β-nucleating agent was also chosen for comparison. The high-pressure crystallization of nucleated PP was carried out under various *P* conditions using a custom-built hydrostatic cell. The crystalline phases present in the samples were identified by wide-angle X-ray diffraction (WAXD). The semi-crystalline morphologies were observed by polarized light microscopy (PLM) in microtomed sections and the melting behavior of pressure-crystallized specimens was studied by differential scanning calorimetry (DSC).

## Experimental

### Materials and sample preparation

A PP grade for injection-molding was provided by Atofina (now Arkema, France) under the reference 3250 MR1 (equivalent to PPH 9081, Total Petrochemicals). Its main molecular parameters are *M*_*n*_ = 42,500 g mol^−1^, *M*_*w*_ = 213,000 g mol^−1^, *M*_*w*_/*M*_*n*_ = 5, and isotacticity index = 0.97. The melt flow index of 25 g (10 min)^−1^ was determined according to the ISO 1133 method under 230 °C/2.16 kg conditions. This polymer was chosen because it produces spherulites easily observable by PLM and was already used in previous works [35–37].

Two nucleating agents known to nucleate efficiently the crystallization of PP in the α-form under *P*_atm_ were selected: Hyperform HPN-20E containing two-third of calcium salt of cis-1,2-cyclohexanedicarboxylic acid and one-third of zinc stearate (as acid scavenger) produced by Milliken Chemical (USA), and poly(tetrafluoroethylene) (PTFE) submicron particles purchased from Polysciences Inc. (Warrington, PA, USA) in the form of aqueous dispersion (DispersEZ-200W2, 30 wt% PTFE content, 200–300 nm particle size), which nucleate efficiently the α-form of PP under *P*_atm_ [38]. The third nucleating agent known to nucleate efficiently the β-form under *P*_atm_ was also used—calcium pimelate (CaPim), synthesized at CMMS, Lodz [8, 39].

PP was mixed with 0.2 wt% of nucleating agents in a Brabender batch mixer at 195 °C, at the speed of 60 rpm for 5 min under N_2_ flow. The nucleated PP samples are denoted as follows: (1) PP/Hyperform-0.2 wt% (PP/H), (2) PP/PTFE-0.2 wt% (PP/T), and (3) PP/CaPim-0.2 wt% (PP/C). To produce a blank control sample, PP without nucleating agent was processed in the same way.

### Crystallization under atmospheric pressure

The efficiency of the nucleating agents was verified under *P*_atm_ by DSC using a TA Instruments DSC 2920 (New Castle, DE, USA), in a N_2_ atmosphere. The specimens were first heated to 250 °C at 10 °C min^−1^, annealed 5 min to erase prior thermal history, and subsequently cooled down to 50 °C at a rate of 10 °C min^−1^. The second heating carried out at 10 °C min^−1^ allowed to examine the melting behavior of the specimens.

### Crystallization under high pressure

**Fig. 1:**
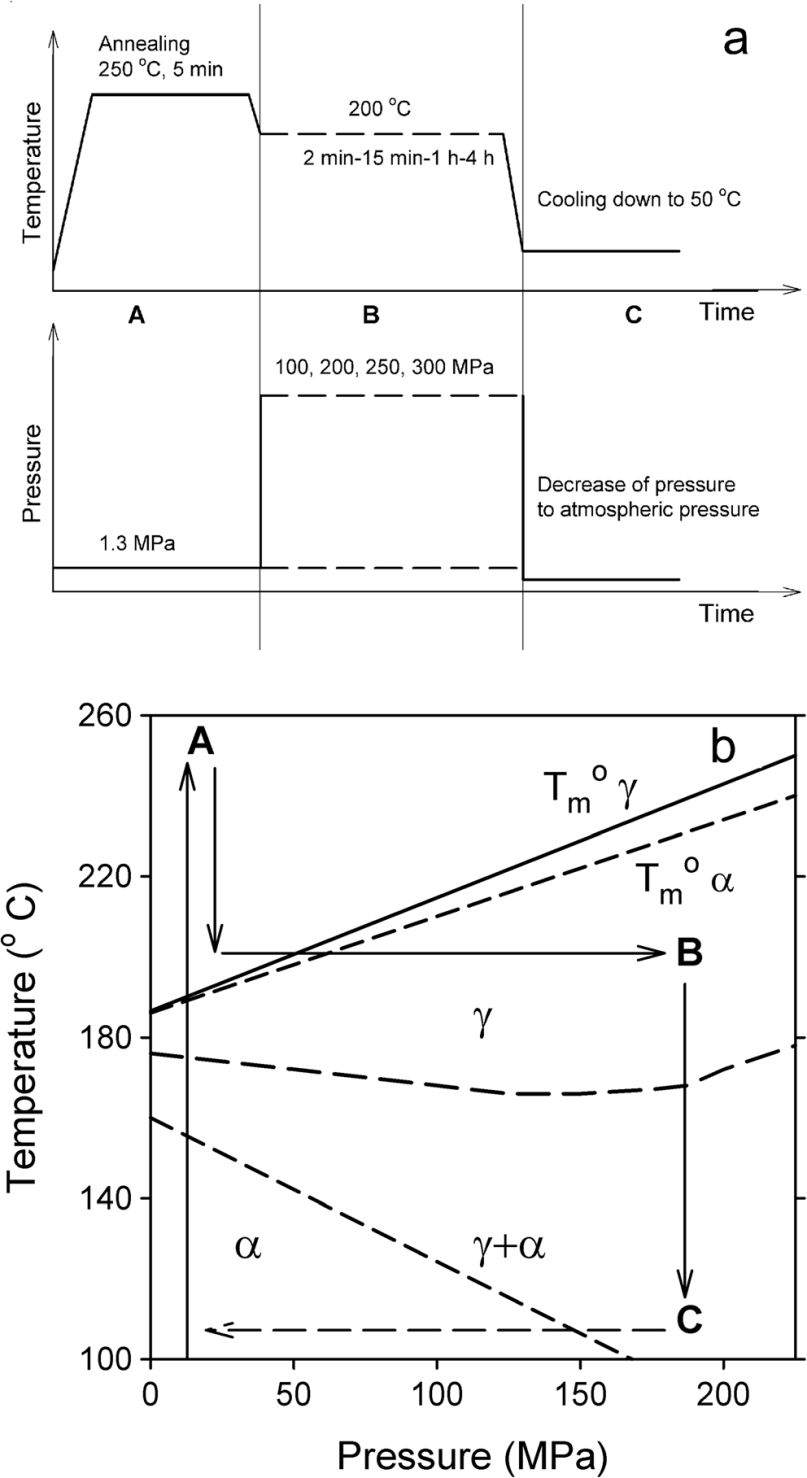
High-pressure crystallization. **a** Scheme of temperature and pressure protocols. **b** Location of the ABC cycle on the phase diagram proposed by Mezghani and Phillips [26]

The high-pressure crystallization of the materials was carried out in a custom-built cell that consisted of a barrel and a piston, described in detail elsewhere [9, 27, 40, 41]. The samples, about 200 mg, were compressed using an Instron tensile testing machine (Instron Corp., High Wycombe, UK), with a cross-head speed of 2 mm min^−1^. The hydrostatic *P* and *T* inside the cell were controlled with an accuracy of ± 0.5 MPa and 1 °C, respectively. The *T* and *P* protocols (Fig. 1a) were the following: (A) heating the specimen up to 250 °C under low external *P* of 1.3 MPa, applied to ensure good thermal contacts, holding it at 250 °C for 5 min and then cooling down to 200 °C; (B) increasing *P* and holding under high *P* at 200 °C with two types of experiments: (1) during 15 min, under various *P*s, 100, 200, 250, or 300 MPa; experiments under low *P* of 1.3 MPa were also conducted for comparison, and (2) under 200 MPa, for various dwell times from 2 min to 4 h, and cooling down to 50 °C, and (C) depressurization. The cooling from 200 °C to 100 °C, during which crystallization could be expected (if not completed at 200 °C), was nearly linear with an average rate of 8 °C min^−1^. According to the phase diagram proposed by Mezghani and Phillips [26], where the ABC cycle has been indicated (Fig. 1b), all the conditions pertain to the γ domain.

It must also be mentioned that although during cooling from 250 to 200 °C, the average cooling rate was close to 8 °C min^−1^, it was decreasing near the target temperature; cooling from 210 to 200 °C required 2–3 min, which is comparable to the shortest dwell time at 200 °C.

### Characterization of crystallized specimens

Our equipment does not allow to follow in situ the isothermal crystallization under high pressure. Therefore, the structures and morphologies of the crystallized specimens were analyzed ex situ using different experimental techniques. The same techniques were applied to examine the specimens crystallized in DSC under *P*_atm_.

The semi-crystalline morphologies were observed on 10-μm-thick microtomed sections by polarized light microscopy (PLM) using a microscope (PZO, Poland) equipped with a video camera.

To characterize their melting behavior, the specimens were heated in DSC to 250 °C at 10 °C min^−1^ under *P*_atm_.

The crystalline structures of the samples were characterized by wide-angle X-ray diffraction (WAXD) in the reflection mode in the 2θ range from 7 to 67 °. A wide-angle goniometer, coupled to a sealed tube X-ray generator (Philips PW3830, Eindhoven, The Netherlands) operating at 30 kV and 50 mA, was used. The X-ray beam consisted of the CuKα radiation (0.154 nm) filtered by a Ni filter and electronically. The slit system used for collecting 2θ scans enabled collection of the diffracted beam with a divergence angle of less than 0.05 °.

**Fig. 2:**
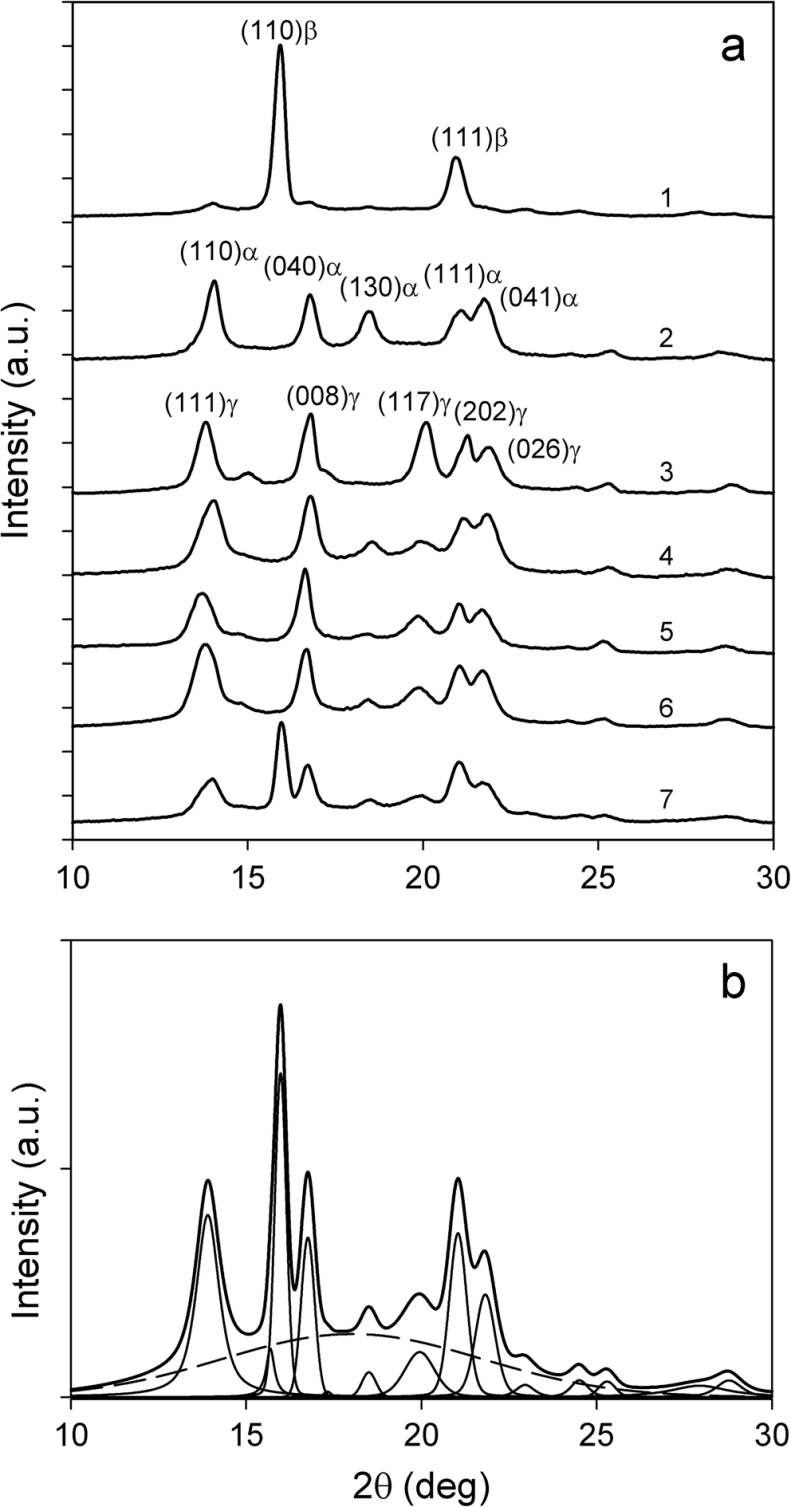
**a** X-ray diffractograms (in 2θ range from 10 to 30 ^o^) of nearly pure β in PP/C formed under 1.3 MPa (1), pure α in PP formed under 1.3 MPa (2), pure γ in PP/T formed under 300 MPa (3), mixtures of α and γ in PP (4), PP/H (5), and PP/T (6) formed under 100 MPa, and mixture of α, β, and γ in PP/C (7) formed under 100 MPa. **b** Deconvolution of (7)

In our experiments, the α, β, and γ phases may be encountered according to the polymer formulation and the crystallization conditions. Each phase is characterized by a typical X-ray diffractogram, as shown in Fig. 2a. Many peaks characteristic of the different phases are located at nearly the same positions. Therefore, only a few well-separated diffraction peaks are available for the identification of the crystallographic forms, when several phases are present as also shown in Fig. 2a. These are (130)_α_, (110)_β,_ and (117)_γ_. It is also necessary to deconvolute the diffraction curve to calculate the proportion of each phase as illustrated in Fig. 2b. This has been done using the WAXSFIT program [42]. The amounts *K*_α_, *Κ*_β_, and *Κ*_γ_ of the α, β, and γ phases in the total crystalline component of PP specimens were determined using the equations given by Turner-Jones et al. [43] for the two-phase case (α + β and α + γ) and by Obadal et al. [44] for the three-phase one.

- For α + β:
  1 $$ \begin{array}{c}\kern1em {K}_{\beta }=I{(110)}_{\beta }{\left[I{(110)}_{\beta }+I{(110)}_{\alpha }+I{(040)}_{\alpha }+I{(130)}_{\alpha}\right]}^{-1}\kern1em \\ {}\kern1em {K}_{\alpha }=1-{K}_{\beta}\kern1em \end{array} $$
- For α + γ:
  2 $$ \begin{array}{c}\kern1em {K}_{\gamma }=I{(117)}_{\gamma }{\left[I{(117)}_{\gamma }+I{(130)}_{\alpha}\right]}^{-1}\kern1em \\ {}\kern1em {K}_{\alpha }=1-{K}_{\gamma}\kern1em \end{array} $$
- For α + β + γ:
  3 $$ \begin{array}{c}\kern1em {K}_{\beta }=I{(110)}_{\beta }{\left[I{(110)}_{\beta }+I{(110)}_{\alpha }+I{(040)}_{\alpha }+I{(130)}_{\alpha }+I{(117)}_{\gamma}\right]}^{-1}\kern1em \\ {}\kern1em {K}_{\gamma }=\left(1-{K}_{\beta}\right)I{(117)}_{\gamma }{\left[I{(117)}_{\gamma }+I{(130)}_{\alpha}\right]}^{-1}\kern1em \\ {}\kern1em {K}_{\alpha }=\left(1-{K}_{\beta}\right)I{(130)}_{\alpha }{\left[I{(117)}_{\gamma }+I{(130)}_{\alpha}\right]}^{-1}\kern1em \end{array} $$

where, for instance, I(117)γ denotes the integral intensity of the (117)_γ_ diffraction peak. The absolute amounts of the α, β, and γ phases can be obtained by multiplying *K*_α_, *Κ*_β,_ and *Κ*_γ_ by the crystallinity *X*_*c*_, which is also deduced from WAXD, the amorphous halos with maxima being also determined by the deconvolution (see Fig. 2b). It has to be mentioned that PP/H samples exhibited orientation with (040)_α_ and/or (008)_γ_ perpendicular to the compression direction (CD). This was most probably caused by orientation of the ruler-like HPN-20E particles by melt flow during compression when the molten polymer filled the cavity of the high-pressure cell. Because of this orientation, PP/H samples were illuminated in CD and in two orthogonal directions perpendicular to CD. Next, the average WAXD curve was calculated for further analysis.

## Results

### Efficiency of the nucleating agents

**Fig. 3:**
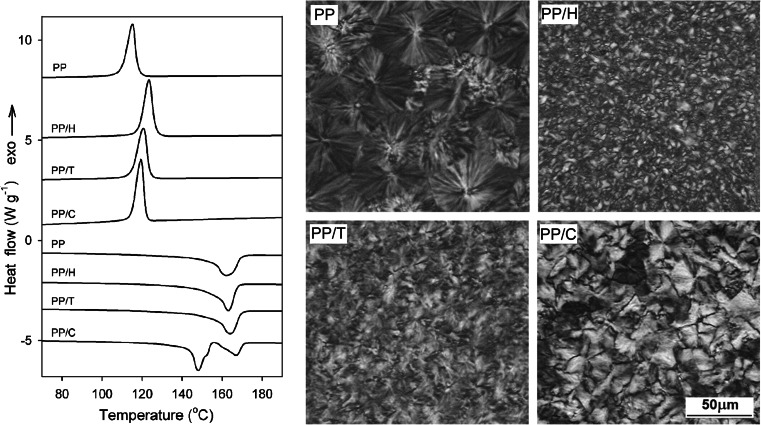
DSC cooling and subsequent heating thermograms and PLM micrographs of thin sections showing structure formed during cooling in PP, PP/H, PP/T, and PP/C. Cooling and heating rate 10 °C min^−1^. In PP/C, a few α-spherulites are observed on the micrograph

Figure 3 allows us to compare the crystallization and melting behavior of the different formulations under *P*_atm_ as well as the structure of the crystallized specimens. The crystallization peak temperatures of PP/H (123 °C) and PP/T (121 °C) are significantly higher than that of neat PP (115 °C). This increase of the crystallization peak temperature is accompanied by a decrease of the spherulite size, particularly in PP/H, evidenced by PLM. These results demonstrate the ability of Hyperform HPN-20E and PTFE DispersEZ-200W2 to enhance the nucleation of the α-form of PP, the strongest effects being obtained with Hyperform HPN-20E. For PP/C, a crystallization peak is centered at 119 °C, whereas PLM reveals the occurrence of the β-phase, with a characteristic spherulitic morphology. The heating thermograms of neat PP, PP/T, and PP/H are featured by single melting peaks centered at 162 °C and 163–164 °C, respectively. This ranking of the melting temperatures (*T*_*m*_) can be correlated to the crystallization temperatures resulting in thicker crystals of the α-phase. For PP/C, two peaks are observed at 167 and 148 °C (the latter with a shoulder), which can be correlated with the presence of the significant amount of the β-phase. Ιt is long known [1] that the β-phase if cooled below 100–110 °C, as in the present case, is susceptible to βα recrystallization followed by melting of the thus formed α-form, which contributes to the high-temperature melting peak.

### Crystalline phases formed under high pressure

The high-pressure cell used for the experiments does not allow us to follow the overall kinetics of isothermal crystallization and to determine when the crystallization is completed. Depending on pressure, dwell time at 200 °C and intensity of primary nucleation, the crystallization could occur during cooling or at isothermal conditions. It is also possible that it started at isothermal conditions and continued during cooling. It is reasonable to believe that, according to the phase diagram shown in Fig. 1, crystallization in the γ-form occurs under high *P* at 200 °C and/or during cooling in the high *T* range, whereas at lower *T* formation of α or β occurs, the latter in PP/C. Epitaxy of γ-lamellae on α-seeds was also observed [e.g., 9] which indicates that a small amount of the α-phase can form in the *T* range of γ-phase formation.

**Fig. 4:**
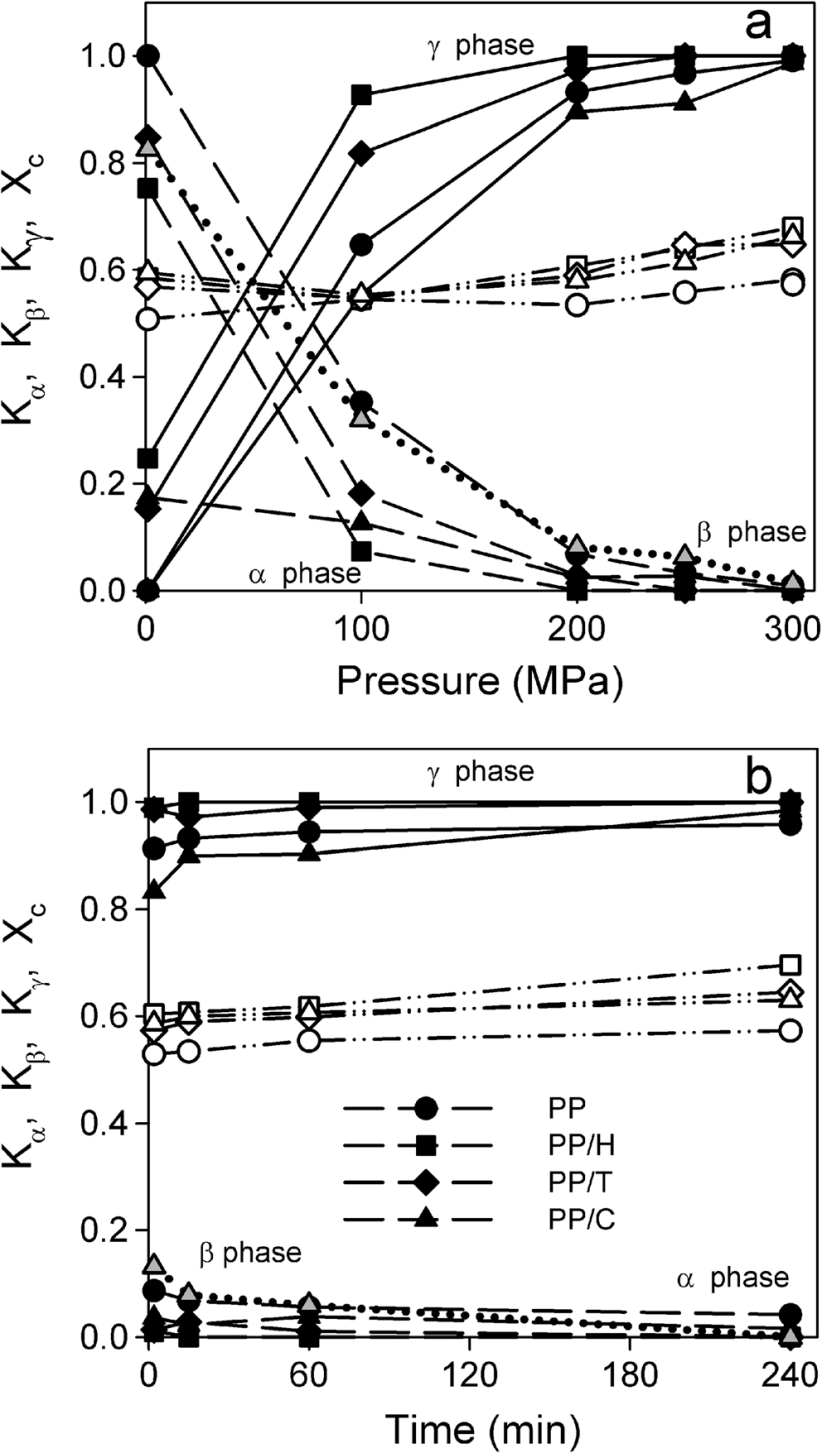
Variations of *K*_α_ (*dashed line*, *black-filled symbols*), *Κ*_γ_ (*solid line*, *black-filled symbols*), *Κ*_β_ (*dotted line*, *gray-filled symbols*), and *X*_*c*_ (*dash-dotted line*, *empty symbols*) in PP, PP/H, PP/T, and PP/C. **a** With the applied pressure for samples held at 200 °C for 15 min. **b** With the dwell time for samples held at 200 °C under 200 MPa

Figure 4a shows the variations of *K*_α_, *Κ*_β_, *Κ*_γ_, and *X*_*c*_ with the applied *P* for the different formulations, after 15 min at 200 °C. Under 1.3 MPa, only the α-phase formed in neat PP; whereas in PP/C, the β-phase formed together with a small fraction of the α-phase. In PP/T and PP/H, a fraction of γ-phase was found together with the predominant α. After 15 min under elevated *P* at 200 °C either α and γ phases or pure γ-phase were found in neat PP, PP/H, and PP/T; most probably α formed during cooling. The γ content increased with the elevation of *P*, which enlarged Δ*T* and accelerated γ crystallization in isothermal conditions. The γ content was the smallest in PP and the highest in PP/H, especially for *P* ≤200 MPa; *K*_γ_ of PP/H reached 1 for *P* = 200 MPa. In PP/C, α, β, and γ phases were found after crystallization under *P* ≤250 MPa. The γ content increased and the β and α contents decreased with increasing *P*, which suggests that the β-phase formed rather during cooling. Contrary to both α-nucleants, the β-nucleant did not enhance crystallization in the γ form. At 300 MPa, all the specimens were essentially composed of the γ-phase, with some cases traces of other phases: α in PP and β in PP/C.

In crystallization experiments at 200 MPa and 200 °C (Fig. 4b), the content of γ-phase depended on dwell time, the most pronounced changes being visible for short times (2–15 min). The γ content in PP/H and PP/T was higher than in neat PP, especially for short dwell times. For PP/H, 15 min at 200 °C was sufficient to reach *K*_γ_ = 1. In PP/C, the γ content increased and the β content decreased with increasing time. The γ amount in PP/C was smaller than in neat PP for short dwell time, but it increased with time and finally became somewhat larger than in neat PP.

It must be mentioned that the differences in *X*_*c*_ are not significant and being of order of few percents are within the limits of experimental error.

### Morphologies

**Fig. 5:**
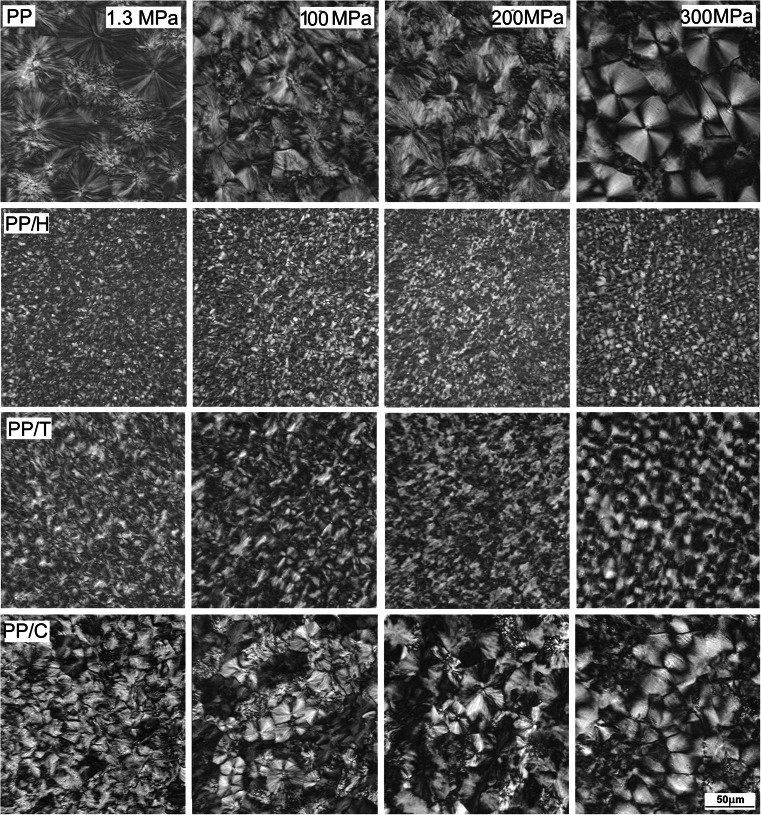
PLM micrographs of thin sections showing structure formed in PP, PP/H, PP/T, and PP/C held at 200 °C for 15 min under various pressures

Figure 5 presents the morphologies developed in samples held at 200 °C during 15 min under various *P*. It illustrates the morphological evolution associated with the transformation of an initial α (neat PP, PP/H, and PP/T) or β (PP/C) predominant phase to a final γ predominant or even pure phase, as deduced from WAXD. In a number of cases, γ-spherulites can be identified by a well-defined Maltese cross [25]. The morphologies formed under 1.3 MPa are similar to those developed during crystallization in DSC under *P*_atm_. Classical α-spherulites are observed in neat PP after crystallization under low *P*. According to the WAXD results, the microstructure for 100 MPa should be a mixture of α and γ-spherulites in comparable numbers. In fact, it is difficult to distinguish the two types of spherulites: if some γ-spherulites can be clearly recognized, it is not easy to identify α ones; most probably, the α-morphologies formed during cooling between existing γ-spherulites. For 200 and 300 MPa, the structure consists of γ-spherulites, with some morphological differences. At 300 MPa, large spherulites with well-identified Maltese cross are observed. For 200 MPa, the spherulites exhibit some axialitic character, possibly due to a smaller Δ*T*. At 300 MPa, some zones containing small spherulites are also visible between the large ones, indicating crystallization during cooling.

For PP/H and PP/T, crystallization at 1.3 MPa results in a very fine microstructure, which is expected to be mainly composed of the α-phase, with already a significant amount of the γ-phase. Such a fine microstructure is also obtained after crystallization at higher *P*, but it is now essentially formed by γ-modification. When applied *P* increases, the γ-phase becomes predominant, with spherulites of similar size, probably nucleated at the same time at 200 °C. However, the spherulites in PP/H are smaller than those in PP/T. Moreover, the same morphological differences are observed in PP/H and PP/T as for neat PP: at 300 MPa spherulites seem to be better ordered.

After crystallization under 1.3 MPa, one observes in PP/C a majority of β-spherulites with a few α-ones. For 100 MPa, three types of spherulites (α, β, and γ) are present, whereas for higher *P*, γ-spherulites are predominant, as can be judged from the WAXD results.

**Fig. 6:**
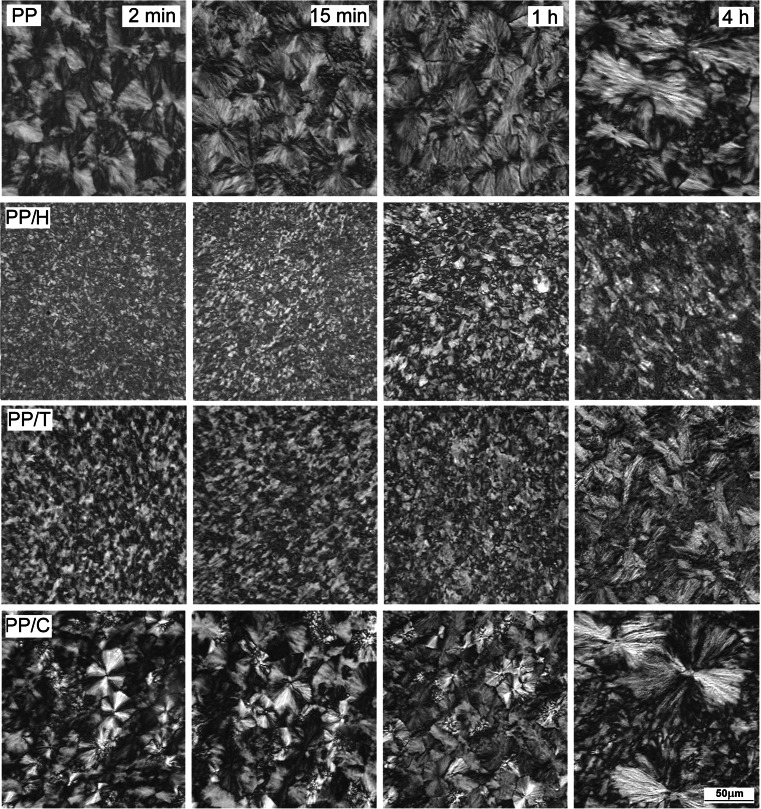
PLM micrographs of thin sections showing structure formed in PP, PP/H, PP/T, and PP/C held at 200 °C under 200 MPa for different times

**Fig. 7:**
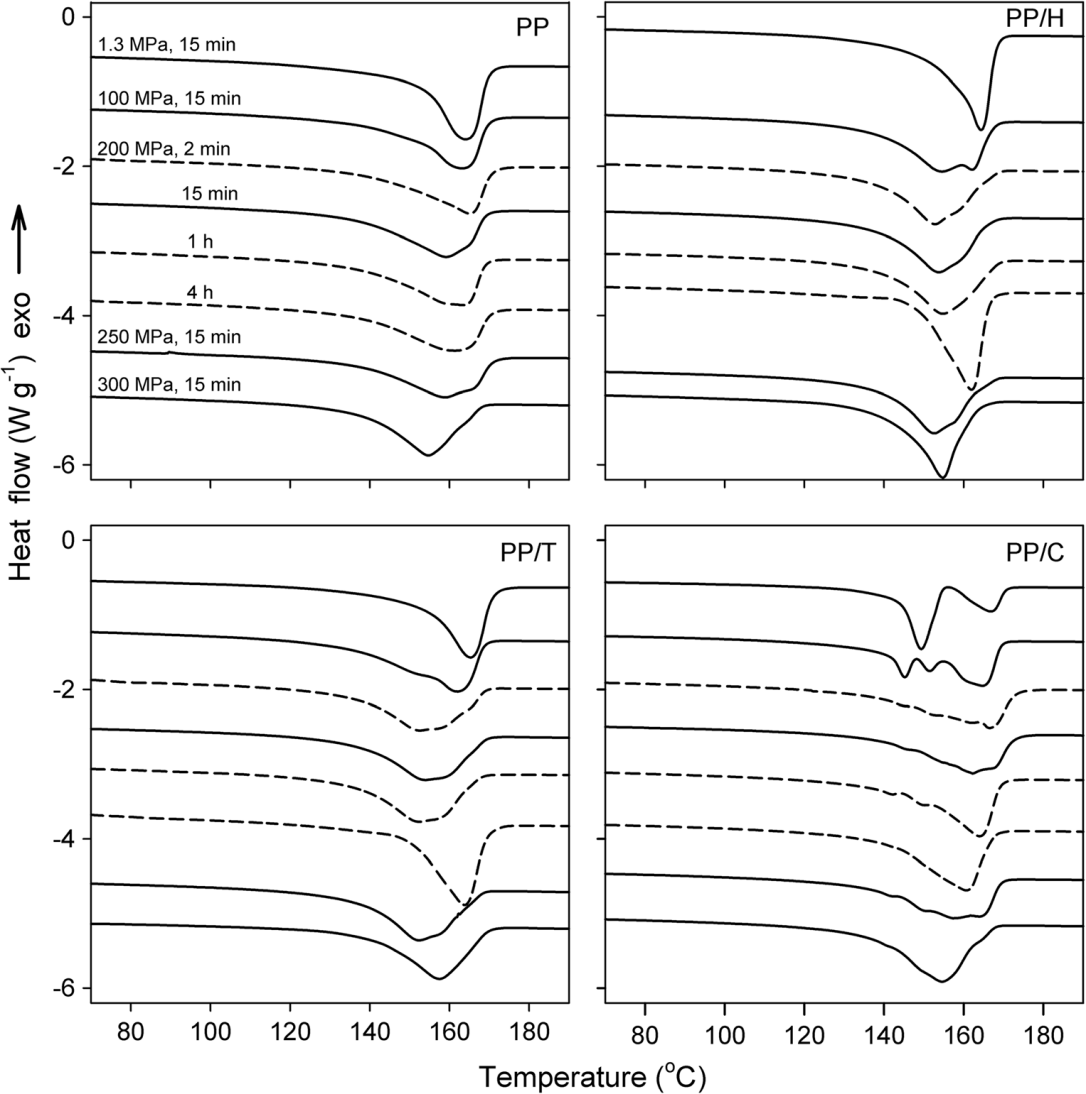
DSC heating thermograms recorded under atmospheric pressure for PP, PP/H, PP/T, and PP/C held at 200 °C for 15 min under various pressures and at 200 °C under 200 MPa for different times

Figure 6 illustrates the influence of the dwell time on the morphologies developed during crystallization at 200 °C under 200 MPa. The microstructures corresponding to 2- and 15-min dwell time are rather similar; larger spherulites are seen in PP, smaller in PP/C, and fine in PP/T and PP/H. Conversely, after 4 h at 200 °C, some large spherulites exhibiting an axialitic character are observed in neat PP and PP/C, accompanied by smaller ones. This is not the case for PP/H and PP/T where the spherulite size is perhaps larger after 4 h but remains homogeneous, with an internal structure that looks coarser. This coarser structure could be due to lamella thickening, which could explain the increase of melting temperature observed in Fig. 7. These results show that for neat PP and PP/C, complete crystallization at 200 °C under 200 MPa requires a longer time. On the contrary, the crystallization of PP/T and PP/H in the same conditions seems to be complete. Moreover, the spherulites in PP/H are markedly smaller than in PP/T.

### Melting behavior

Figure 7 presents the melting curves of the specimens crystallized at various conditions. The melting behavior of specimens crystallized under 1.3 MPa is similar to that of the materials crystallized in DSC for the already given reasons.

For neat PP, PP/H, and PP/T, one globally notes the evolution from the peak of the α-phase (1.3 MPa) to that of the γ-phase (300 MPa). The peaks of the α-phase are located at 164–165 °C (neat PP, PP/H, PP/T). For samples annealed under *P* between 100 and 250 MPa, broad peaks are observed, some of them exhibiting a shoulder, which can result from several causes: (i) residual α-phase, especially for 100 MPa, that contributes to the high-temperature part of the peak and (ii) a population of crystals appeared during cooling, that melt at lower *T*. The *T*_*m*_s of the γ-phase after crystallization under 300 MPa are 155 °C for neat PP and PP/H; whereas at 157.5 °C for PP/T, the *T*_*m*_s of γ-phase are generally smaller than those of the α one. As discussed by Mezghani and Phillips [26], the DSC curves for 300 MPa actually represent the melting of the γ-form rather than the transformation of the γ to the α-form.

The thermal behavior of PP/C is rather complicated. After crystallization under 1.3 MPa, similarly as under *P*_atm_, two peaks are observed at 149 and 167 °C, respectively, with α to β enthalpy ratio approx. 25:75, which is larger than the α to β content ratio determined by WAXD. As discussed above, these results most probably from βα recrystallization followed by melting of the thus formed α-phase. At 100 MPa, the melting behavior of PP/C is even more complicated with a composite endotherm reflecting the coexistence of three crystalline phases (α, β, γ), in agreement with WAXD and microscopic observations. In addition to the two peaks at *T*_*m*_ *s* more or less equal to those observed for the specimens crystallized under *P*_atm_ in DSC and under 1.3 MPa, the third peak appeared at 145 °C. Moreover, the peak at the highest *T*_*m*_ of 165 °C, exhibited a low temperature shoulder, which reflects the melting of the γ-phase. Yi et al. [45] reported the appearance of an additional melting peak of β-crystals between 140 and 150 °C for β-nucleated PP crystallized at large Δ*T*. Hence, the peak at 145 °C can be attributed to melting of β-crystals formed during cooling but, owing to higher *P*, at larger Δ*T* than those in samples crystallized under *P*_atm_ or 1.3 MPa.

Further increase of *P* (and Δ*T*) increases the γ content and decreases the β content. Then, the melting peaks recorded for crystallization under 200 and 250 MPa are broad and complex, which may originate from different causes: isothermally and non-isothermally crystallized γ-phase and residual β-phase crystallized during cooling (including possible βα recrystallization). Finally, for 300 MPa, the DSC trace may be at first glance described as a single peak at 154.5 °C, associated with isothermally crystallized γ-phase. However, a careful examination reveals changes in slope possibly associated with small quantity of β-phase, as well as with some γ-phase crystallized during cooling.

Moreover, Fig. 7 shows also the melting curves of the specimens crystallized at 200 °C and 200 MPa for different times. One globally notes the evolution from the α (PP, PP/H, and PP/T) and the α + β phases (PP/C) to the γ-phase, due to the increase of dwell time at the conditions pertaining to the formation of γ-phase. Even in the final stage, some slight differences can be detected. In PP, some significant amount of the residual α-phase could explain broader melting peaks. On the contrary, PP/H and PP/T seem to be constituted only by the γ-phase, with a homogeneous microstructure exhibiting some evidence of lamella thickening, which results in a narrower melting peak, a higher *T*_*m*_ (162–164 °C).

## Discussion

### α-nucleating agents

Two α-nucleating agents have been considered in this work. Hyperform HPN-20E is a commercial product, which nucleates both polyethylene and polypropylene. It is a carboxylic acid salt and belongs to the category of particulate nucleating agents (see Introduction), which remains in the solid state in the melting-crystallization procedures of PP. As shown in Fig. 2, it is a very efficient α-nucleating agent under *P*_atm_. PTFE, which belongs to the category of polymeric nucleating agents, is less efficient than HPN-20E. PTFE fibers, films, and particles are known to nucleate PP crystallization in the α-phase [38, 46–50].

After crystallization under *P*_atm_ or 1.3 MPa, only the α-modification formed in neat PP, whereas in PP/T and PP/H a significant fraction of γ-phase was found together with predominant α. This is in agreement with the results of Foresta et al. [24], who showed that classical α-nucleants such as talc or dibenzylidene sorbitol (DBS) enhanced the formation of some γ-phase even under *P*_atm_. In the present study after crystallization under elevated *P*, α and γ were found in neat PP, PP/H, and PP/T; most probably α formed during cooling. The γ content in PP/H and PP/T was higher than in neat PP, especially for moderate *P* (100 MPa, 200 °C, 15 min) or for short time, up to 1 h, of crystallization at 200 MPa and 200 °C. Therefore, the presence of HPN-20E and PTFE particles enhances crystallization of PP in the γ-form under elevated *P*. Two mechanisms can be envisaged: (i) direct nucleation of the γ-form on the substrate or (ii) nucleation of α seeds, on which the γ-form then grows. Within the framework of the epitaxy theory, nucleation of PP in the α-phase mainly involves the (010)_α_ plane, with substrates matching a periodicity of about 4.2, 5, or 6.6 Å in the (010)_α_ plane. This family includes benzoic acid and its salts, as well as polyethylene. This epitaxy also applies for the γ form and involves the equivalent (001)_γ_ plane [51]. It could also concern HPN-20E, which belongs to the same category as benzoic acid and its salts (hypothesis (i)). On the contrary, α crystallization on PTFE substrate is related to a specific epitaxy involving the (110)_α_ plane and a 5.5–5.6 Å periodicity, which does not apply for the γ-form [49]. Therefore, the following scenario can be proposed: the α-phase nucleates first on the PTFE substrate, and then the crystallization continues in the γ-form through the conventional γ/α epitaxy [52].

An important thermodynamic effect of *P* is increase of the *T*_*m*_^0^. For the α-phase, the following pressure dependence of *T*_*m*_^0^ has been proposed [53]:

4 $$ {T_m}^0(P)={T_m}^0\left({P}_{atm}\right)+0.283P-2.08\ {10}^{-4}{P}^2 $$

with *P* in MPa. *T*_*m*_^0^(*P*_*atm*_) = 208 *°*C has been retained for the PP under investigation [37].

For our PP, under 100–300 MPa *T*_*m*_^0^(*P*) is in the range of 234–274 °C which corresponds to *ΔT* = *T*_*m*_^0^(*P*) − *T*_*c*_ from 34 to 74 °C for *T*_*c*_ = 200 °C. The corresponding *T*_*c*_s under *P*_atm_ would be 174–134 °C. Let us recall that under *P*_atm_, the complete crystallization of neat PP takes about 80 min at 130 °C [32]. Of course, these considerations concern only the α-phase, but according to Fig. 1, the *T*_*m*_^0^ of the γ-phase is supposed to be from 5 to 10 °C higher than the one of the α-phase, which increases the undercoolings.

The above calculations show that even taking the higher value of *T*_*m*_^*o*^ than that determined by Phillips and Mezhgani [26] and in the presence of nucleating agents, crystallization cannot take place at 200 °C under 100 MPa, and of course under 1.3 MPa, but necessarily during cooling, with the help of nucleating agents for PP/H and PP/T, which leads to a smaller spherulite size with respect to neat PP. In the higher *T* range, crystallization occurs in the γ-form, which is more stable in this range, and then in the α one at lower *T*s. This explains the increase of the γ content in α-nucleated formulations. Micrographs of PP/T and PP/H crystallized at 200 and 300 MPa show a homogeneous distribution of spherulites, smaller than in neat PP, indicating that the nucleated agent has played its role during isothermal crystallization at 200 °C. Now the question is: is the crystallization completed at 200 °C under 200 and 300 MPa? Broad melting peaks obtained at 200 and 250 MPa (Fig. 7) show that a significant part of the crystallization has occurred during cooling. Conversely, the melting peak obtained for 300 MPa is better defined and shows that most of the crystallization happened in isothermal conditions.

This analysis is supported by the experiments at 200 °C and 200 MPa. After 4 h of crystallization, some large spherulites exhibiting an axialitic character are observed in neat PP. This is not the case for PP/H and PP/T where the spherulite size is perhaps larger after 4 h of crystallization but remains homogeneous and much smaller than in neat PP, showing the activity of the nucleants. Furthermore, the internal structure of the spherulites looks coarser, which could be due to lamella thickening. It could explain the narrower melting peaks with higher *T*_*m*_ in Fig. 7. However, no significant increase of crystallinity is observed in Fig. 4b and Fig. 7.

### β-nucleating agent

CaPim has been recognized as a nucleating agent for the β-phase of PP by Li and Cheung [54] and Varga et al. [55] and was used in previous works [8, 39, 56]. The nucleating activity of β-nucleants has been related to an epitaxy involving a 6.5–6.6 Å periodicity. For some nucleating agents, for instance γ-quinacridone, this epitaxy is also possible for the α-phase [57].

As for the other formulations, crystallization does not take place at 200 °C under 100 MPa, and of course under 1.3 MPa, but during cooling. Obviously, the β-nucleant does not enhance crystallization in the γ-phase, since the γ content is even lower than in neat PP. The γ content increases and the β content decreases with increasing *P*, a more and more important part of the crystallization occurring at 200 °C in isothermal conditions. For 300 MPa, there is only a trace of β-phase and the γ content is about the same as in neat PP. The γ content in PP/C is smaller than in neat PP for short annealing time at 200 °C and 200 MPa, but it increases with time and finally becomes higher after 4 h of isothermal crystallization. This is correlated to the decrease of the α content down to zero and the β content to very low value. Conversely, in neat PP it remains a certain amount of α. Note that the final morphologies (200 °C, 300 MPa, 15 min and 200 °C, 200 MPa, 4 h) of PP and PP/C are close to each other. All these facts show that the β nucleating agent has only a slight effect on the crystallization in the γ-phase.

Only few articles have considered the respective effects of high pressure and β-nucleating agents [44, 58]. Obadal et al. [44] have tested NU 100 (*N*,*N*′-dicyclohexylnaphthalene-2,6-dicarboxamide) as β-nucleant and concluded that it enhances the formation of the β-phase at lower *P* and also at high *P* but at low *T*. Even in β-nucleated PP high *P* favors the formation of the γ-phase whereas the formation of the α-phase is supported by low *P* and high *T*. Although the range of *T* investigated is not the same, these statements are consistent with our own results.

Recently, Yang et al. [58] have also used another very efficient commercial β-nucleant: TMB-5, an aryl amide derivative, which has a chemical structure similar to NU 100. Some of their results are similar to ours, i.e., an increase of the γ content and a decrease of the β content with increasing *P*. Nevertheless, there exist some significant differences: for 116 MPa the γ content is higher than in neat PP, and the morphology is deeply modified by nucleation. The authors propose a model in which there is first crystallization of α and β lamellae on TMB-5 needles. Then, the γ-phase develops from α-seeds, thus limiting and even suppressing the crystallization in the β-phase. Thus, according to the type of β-nucleant, the behavior may be very different.

## Conclusion

The presence of α-nucleating agents efficiently enhanced crystallization of PP in the γ-form under high *P*, both during isothermal crystallization and during cooling. These nucleation effects of HPN-20E and PTFE can be produced either by direct nucleation of the γ-form, or through nucleation of α-seeds, on which the γ-form grows. PTFE nucleates the α-form through epitaxy involving (110)_α_ crystallographic planes; this epitaxy does not apply for the γ-phase. Hence, in the case of PTFE, the second mechanism seems to be more probable. The γ-nucleation ability of the α-nucleating agents results in a synergistic effect of their presence and high *P* on crystallization of PP in the γ-form.

The β-nucleating agent, CaPim, very weakly enhances crystallization of PP in the γ-form under high *P*. CaPim is inefficient in nucleation of the γ-form; and as a result, there is a competition between its nucleating activity and high *P*. Under sufficiently high *P*, the role of CaPim becomes inconspicuous and the crystallization of PP is mainly determined by high *P*.

## References

[1] Varga J, Karger-Kocsis J (1993). Polypropylene: structure, blends and composites, vol 1.

[2] Moitzi J, Skalicky P (1993). Polymer.

[3] Lovinger AJ, Chua JO, Gryte CC (1977). J Polym Sci Polym Phys Ed.

[4] Pawlak A, Piorkowska E (2001) Colloid Polym Sci 279:939–946

[5] Bruckner S, Meille SV (1989). Nature.

[6] Meille SV, Bruckner S, Porzio W (1990) Macromolecules 23:4114–4121

[7] Bruckner S, Meille SV, Sozzani P, Torri G (1990). Makromol Chem Rapid Commun.

[8] Lezak E, Bartczak Z, Galeski A (2006). Polymer.

[9] Lezak E, Bartczak Z, Galeski A (2006). Macromolecules.

[10] Ferro DR, Bruckner S, Meille SV, Ragazzi M (1992). Macromolecules.

[11] Meille SV, Ferro DR, Bruckner S (1992). Polym Prepr (ACS, Div Polym Chem).

[12] Morrow DR, Newman BA (1968). J Appl Phys.

[13] Addink EJ, Beintema J (1961). Polymer.

[14] Lotz B, Graff S, Wittmann JC (1986). J Polym Sci Part B: Polym Phys Ed.

[15] Kojima M (1967). J Polym Sci Part B: Polymer Lett.

[16] Turner-Jones A (1971). Polymer.

[17] Alamo RG, Kim MH, Galante MJ, Isasi JR, Mandelkern L (1999). Macromolecules.

[18] Guidetti GP, Busi P, Giulianelli I, Zanetti R (1983). Eur Polym J.

[19] Busico V, Corradini P, De Rosa C, Di Benedetto E (1985). Eur Polym J.

[20] Avella M, Martuscelli E, Della Volpe G, Segre A, Rossi E, Simonazzi T (1986). Makromol Chem.

[21] Marigo A, Marega C, Zanetti R, Paganetto E, Canossa E, Coleta F, Gottardi F (1989). Makromol Chem.

[22] Mezghani K, Phillips PJ (1995). Polymer.

[23] Hosier IL, Alamo RG, Lin JS (2004). Polymer.

[24] Foresta T, Piccarolo S, Goldbeck-Wood G (2001). Polymer.

[25] Mezghani K, Phillips PJ (1997). Polymer.

[26] Mezghani K, Phillips PJ (1998). Polymer.

[27] Zapala K, Piorkowska E, Hiltner A, Baer E (2012). Colloid Polym Sci.

[28] Beck HN (1967). J Appl Polym Sci.

[29] Binsbergen FL (1970). Polymer.

[30] Binsbergen FL (1973). J Polym Sci Polym Phys Ed.

[31] Wittmann JC, Lotz B (1981). J Polym Sci Polym Phys Ed.

[32] Wittmann JC, Lotz B (1990). Prog Polym Sci.

[33] Fillon B, Wittmann JC, Lotz B, Thierry A (1993). J Polym Sci Part B: Polym Phys Ed.

[34] Gahleitner M, Grein C, Kheirandish S, Wolfschwenger J (2011). Int Polym Process.

[35] Monasse B, Smirnova J, Haudin JM, Chenot JL (2004). Int Polym Process.

[36] Boyer SAE, Grolier JPE, Yoshida H, Haudin JM, Chenot JL (2009). J Mol Liq.

[37] Boyer SAE, Robinson P, Ganet P, Melis JP, Haudin JM (2012). J Appl Polym Sci.

[38] Masirek R, Piorkowska E (2010). Eur Polym J.

[39] Lezak E, Bartczak Z (2005). Fibres Text Eastern Eur.

[40] Psarski M, Galeski A, Piorkowska E (2000). Macromolecules.

[41] Kazmierczak T, Galeski A, Argon AS (2005). Polymer.

[42] Rabiej M, Rabiej S (2006). Analiza rentgenowskich krzywych dyfrakcyjnych polimerow za pomoca programu komputerowego WAXSFIT.

[43] Turner-Jones A, Aizlewood JM, Beckett DR (1964). Makromol Chem.

[44] Obadal M, Čermák R, Stoklasa K (2005). Macromol Rapid Commun.

[45] Yi QF, Wen XJ, Dong JY, Han CC (2008) Polymer 49: 5053–5063

[46] Fitchmun DR, Newman S (1970) J Polym Sci A2 Polym Phys 8:1545–1564

[47] Wang C, Hwang LM (1996). J Polym Sci B Polym Phys.

[48] Wang C, Liu CR (1999). Polymer.

[49] Yan S, Katzenberg F, Petermann J, Yang D, Shen Y, Straupé C, Wittmann JC, Lotz B (2000). Polymer.

[50] Gadzinowska K, Piorkowska E (2003). Polimery.

[51] Mathieu C, Thierry A, Wittmann JC, Lotz B (2000). Polymer.

[52] Lotz B, Graff S, Straupé C, Wittmann JC (1991). Polymer.

[53] Fulchiron R, Koscher E, Poutot G, Delaunay D, Régnier G (2001). J Macromol Sci - Phys.

[54] Li JX, Cheung WL (1997) J Vinyl Addit Technol 3:151–156

[55] Varga J, Mudra I, Ehrenstein GW (1999). J Appl Polym Sci.

[56] Lezak E, Bartczak Z (2008). J Polym Sci B Polym Phys.

[57] Mathieu C, Thierry A, Wittmann JC, Lotz B (2002). J Polym Sci B Polym Phys.

[58] Yang G, Li X, Chen J, Yang J, Huang T, Liu X, Wang Y (2012). Colloid Polym Sci.

